# Elucidating the network interactions between 21 secreted *Mycobacterium tuberculosis* proteins and host proteins: the role of DnaK in enhancing *Mtb* survival via LDHB: Network interactions between 21 secreted *Mtb* proteins and host proteins

**DOI:** 10.3724/abbs.2024041

**Published:** 2024-03-26

**Authors:** Hong Chen, Xiang He, He-Wei Jiang, Yun-Xiao Zheng, Hai-Nan Zhang, Fan-Lin Wu, Zhao-Wei Xu, Shu-Juan Guo, Sheng-Ce Tao

**Affiliations:** 1 Shanghai Center for Systems Biomedicine Key Laboratory of Systems Biomedicine (Ministry of Education) Shanghai Jiao Tong University Shanghai 200240 China; 2 College of Basic Medical Science in Shanghai Jiao Tong University Shanghai Jiao Tong University Shanghai 200025 China; 3 Lin Gang Laboratory Shanghai 201306 China; 4 Institute of Neuroscience Chinese Academy of Sciences Shanghai 200031 China; 5 Key Laboratory of Molecular Module-Based Breeding of High Yield and Abiotic Resistant Plants in Universities of Shandong School of Agriculture Ludong University Yantai 264025 China; 6 School of Basic Medical Sciences in Fujian Medical University Fujian Medical University Fuzhou 350112 China

Among infectious diseases, *Mycobacterium tuberculosis* ( *Mtb*) infection remains the world’s leading cause of death, at ~1.5 million in 2022. Approximately one-quarter of the global population is infected with *Mtb*, with more than 10 million new infections occurring annually ( https://www.who.int/publications/i/item/9789240083851). Although antibiotics target essential biological processes of *Mtb*, their overuse has led to an increase in the prevalence of antibiotic-resistant strains, especially multidrug-resistant (MDR) and extensive drug-resistant (XDR) strains [1]. This underscores the urgent need for new drug targets to effectively combat *Mtb*. *Mtb* is an adept intracellular pathogen that has evolved strategies to evade hostile environments within host cells, such as macrophages [2]. It utilizes well-regulated secretion systems to transport proteins across the cytoplasmic membrane or through the cell wall, making secretory proteins promising drug or vaccine targets [3]. *Mtb* employs several secretion pathways, including the conserved Sec secretory pathway, the twin-arginine translocation (TAT) pathway, and a specialized Type VII secretion system, also known as the ESX system [3]. It secretes a vast array of proteins that function as effectors in various roles. A previous study identified approximately 1314 proteins in the culture filtrate of *Mtb* [4], highlighting the important roles of these secreted proteins in virulence and immune evasion. While some mycobacterial secreted proteins are known to modulate the host immune response, many remain uncharacterized. Therefore, elucidating the interactions between *Mtb*-secreted proteins and human proteins is vital for understanding *Mtb* pathogenesis and could pave the way for the development of novel drug targets.

Potential interactions between secreted proteins of *Mtb* and host proteins were previously identified using a human proteome microarray [5]. By integrating these data with additional crucial *Mtb*-secreted proteins from various studies [ 6, 7], we compiled a list of 21 candidate *Mtb* proteins. We expressed *Mtb* proteins in HEK293T cells. The open reading frames (ORFs) of these *Mtb* proteins were inserted into a eukaryotic expression vector, pCMV3-C-FLAG, which contains a 3× Flag tag at the C-terminus, followed by confirmation of expression via anti-FLAG western blot analysis. Cell lysates overexpressing *Mtb* proteins were collected approximately 48 h posttransfection for immunoprecipitation (IP) using anti-Flag antibodies to enrich for Flag-tagged *Mtb* proteins and their interacting host proteins, followed by identification through mass spectrometry (MS).

To enhance the reliability of the identified interacting proteins, we utilized SILAC (Stable Isotope Labelling by Amino Acids in Cell Culture) [8] for relative quantitative MS. In the SILAC experiments, two cell populations were differentiated: one was transfected with the vector and grown in medium containing ‘Light’ (normal) lysine and arginine, and the other was transfected with *Mtb* ORFs and grown in medium containing ‘Heavy’ lysine (89988; Thermo Fisher Scientific, Waltham, USA) and arginine (89210; Thermo Fisher Scientific), which included ^13^C instead of ^12^C, resulting in a 6 Da mass shift in peptides incorporating heavy ^13^C _6_-Arg and ^13^C _6_-Lys compared to those with light ^12^C _6_-Arg and ^12^C _6_-Lys.

Lysates from the heavy and light groups were mixed and then subjected to enrichment of interacting proteins using Protein G beads (10003D; Thermo Fisher Scientific) and a Flag antibody (F1804; Sigma, St Louis, USA). The workflow is detailed in Figure 1A. A comparison with proteins identified in previous studies [9] demonstrated high confidence, as depicted in Figure 1B, with a *P* value for overlapping proteins less than 1.8×10 ^–5^ and an enrichment factor of 5.93. The overlapping proteins included SARNP, TIMM13, TXNRD1, NUP153, AHCYL1, BTF3, NAP1L4, MCM7, NUDT21, and H3-3A. The 21 secreted *Mtb* proteins span seven of the eleven categories defined by the tuberculosis database, with the largest number associated with cell wall and cell processes, as illustrated in Figure 1C.

**Figure 1:**
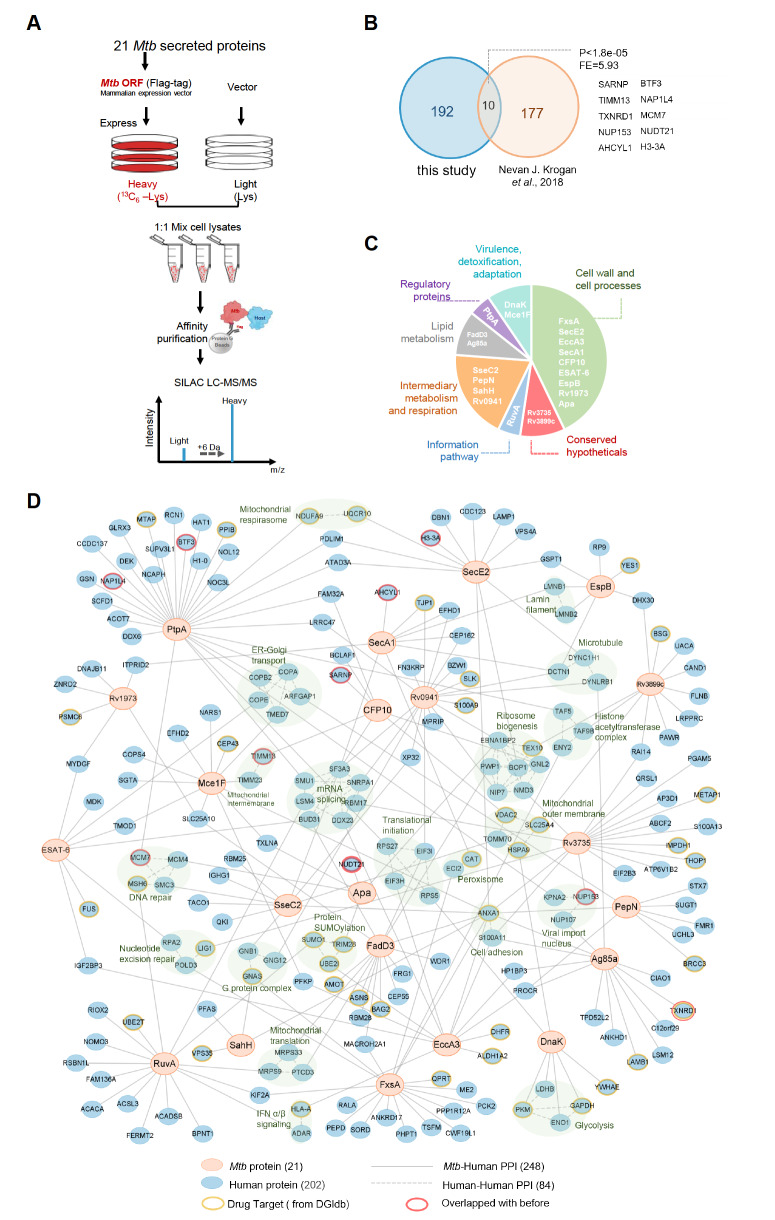
Figure 1 Construction of an interaction network between secreted Mtb proteins and human proteins (A) Comparison of proteins identified in this study with those identified in previous studies. The significance of overlap was assessed using the hypergeometric test (refer to Supplementary Table S1 for details). (B) Functional categorization of Mtb-secreted proteins, as listed in the tuberculosis database (https://mycobrowser.epfl) (for further details, see Supplementary Table S2). (C) Development of the Mtb-human protein-protein interaction (PPI) network. This network features 21 Mtb-secreted proteins represented by orange nodes and 202 human proteins represented by blue nodes (additional information can be found in Supplementary Table S3). Drug targets are highlighted with yellow circles, while proteins overlapping with prior studies are marked with red circles. The solid lines represent direct Mtb-human protein interactions, whereas the dotted lines show interactions among human proteins. High-confidence human protein interaction data were obtained from the STRING database.

Then we constructed a network of interactions between *Mtb*-secreted proteins and human proteins, as shown in Figure 1D. Cytoscape (version 3.10) was used to construct the interaction network. The network comprises 21 *Mtb*-secreted proteins (orange), 202 human host proteins (blue), and 248 edges representing *Mtb*-human protein interactions (solid gray lines). Eighty-four human protein-protein interactions were identified from the STRING database under high-confidence conditions (dashed gray lines). Existing drug targets from DrugBank ( https://go.drugbank.com/) are highlighted with yellow lines for repurposing the existing drug. Proteins overlapping with previous studies are marked with red lines. Enrichment analysis of human proteins was performed by PANTHER ( https://www.pantherdb.org/). The enrichment terms were determined by Fisher’s exact tests. Significant enrichment terms with a fold enrichment>2 and a false discovery rate-corrected *P* value<0.05 were obtained, as indicated by the shaded green area in Figure 1D. The host biological processes associated with each *Mtb-*secreted protein are shown in Figure 2A.

**Figure 2:**
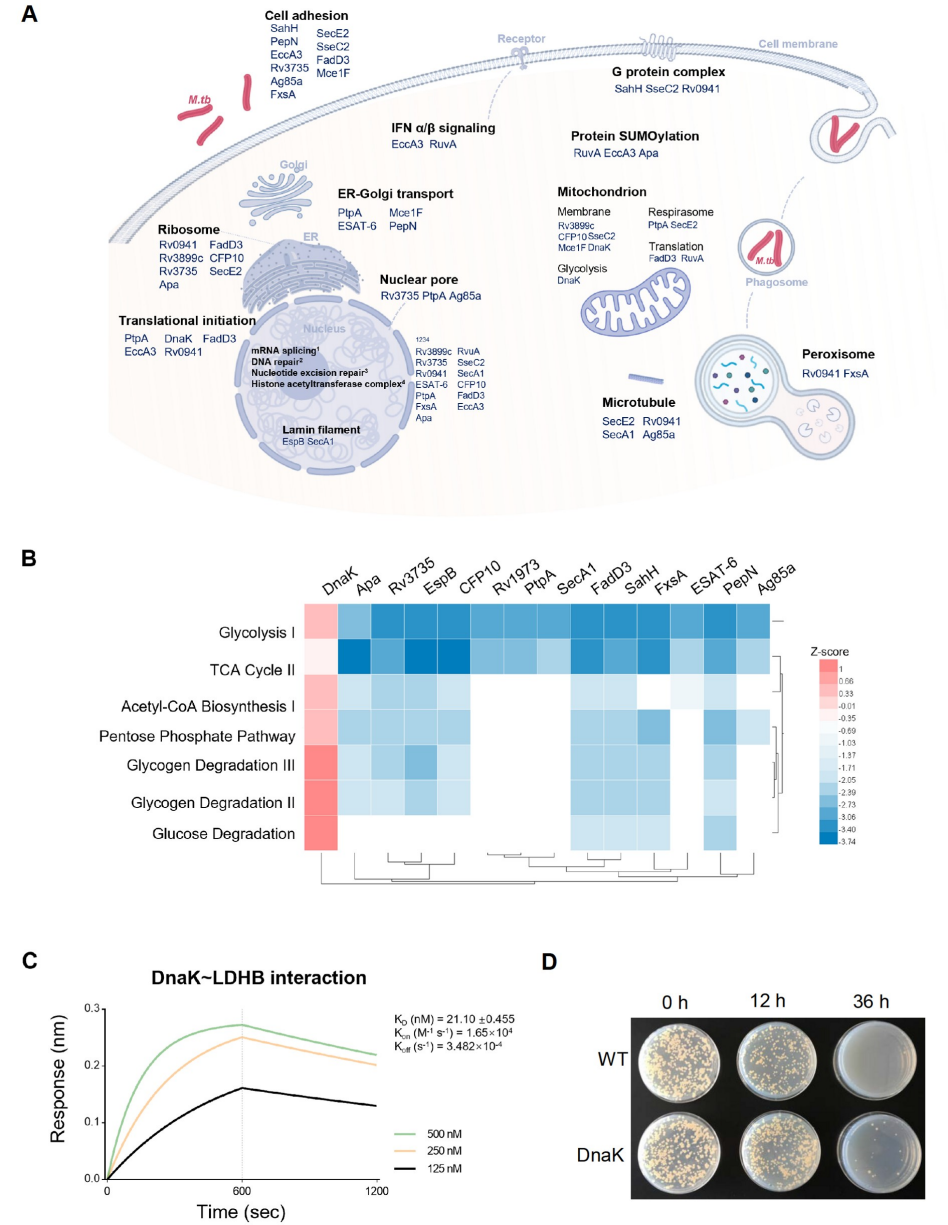
Figure 2 DnaK enhances Mtb growth in macrophages through host glucose metabolism (A) Schematic diagram of the Mtb infection process in host cells and the predicted functions of each Mtb protein derived from GO enrichment terms. (B) Heatmap representing glucose metabolic pathways enriched by human proteins that changed significantly (ratio ≥ 1.5 or ratio ≤ ‒1.5) after overexpression of the Mtb protein (see Supplementary Table S4). The Z scores are shown by color. (C) The affinity constant (KD) of Mtb DnaK for human LDHB was approximately 21 nM. Biolayer interferometry (BLI) was performed to determine the affinity constant. The protein concentration ranged from 500 nM to 125 nM. (D) DnaK Enhances Mtb Survival within Macrophages. The survival of the H37Ra strains in macrophages (THP-1 cells activated with PMA) was assessed after 0, 12, and 36 h. The upper line represents wild-type H37Ra strains, while the lower line shows H37Ra strains with DnaK overexpression.

The schematic diagram in Figure 2A, created using Biorender ( https://www.biorender.com/), illustrates the process of *Mtb* infection. In the diagram, cell processes are denoted in black font, *Mtb* proteins are denoted in blue, and *Mtb* itself is represented by red rod-shaped patterns. During infection, macrophages form phagosomes that subsequently merge with peroxisomes to eliminate intracellular *Mtb*. Our study revealed interactions between the *Mtb* proteins Rv0941 and FxsA and the peroxisomal proteins ECL2 and CAT, highlighting a mechanism by which *Mtb* influences host cellular processes. Additionally, *Mtb* proteins also engage with cell membrane receptors to activate downstream signaling pathways, including host immune responses such as the interferon pathway, as evidenced by interactions between EccA3, RuvA, and the IFNα/β signaling proteins HLA-A and ADAR.

Furthermore, we discovered that *Mtb* mediates cell adhesion through ANXA1 and S100A11 and interacts with G protein complexes via GNB1, GNG12, and GNAS. *Mtb* also impacts host gene expression-related pathways, including those related to mRNA processing, translation, ribosome function, and ER-Golgi transport, through COPB1, COPE, TMED7, COPA, and ARFGAP1. These interactions suggest that *Mtb* can evade host immune responses and maintain intracellular survival by manipulating specific pathways.

Notably, our findings indicate that mitochondria-mediated metabolic pathways are significantly affected by *Mtb* proteins. *Mtb* proteins such as Rv3899c, CFP10, Mce1F, PtpA, SecE2, FadD3, RuvA, and DnaK were found to interact with mitochondria-related pathways, including those involving the mitochondrial membrane, the respirasome, translation, and glycolysis. This finding underscores the extensive influence of *Mtb* on host cellular mechanisms and metabolic processes.

Global protein changes were also identified by SILAC MS in cell lysates overexpressing *Mtb* proteins. Proteins with heavy/light ratios >2 were extracted, and enrichment analysis was performed using Ingenuity Pathway Analysis (IPA) software (QIAGEN, Hilden, Germany). The heatmap shows the comparative results with the Z score [Z=(x‒μ)/σ], as depicted in Figure 2B.

Biological process analysis revealed that DnaK significantly upregulates host glycan metabolism, including glycolysis, the TCA cycle, and glycogen degradation. Among the seven proteins that interact with DnaK, four are involved in glycolysis. We hypothesize that DnaK might affect *Mtb* growth by utilizing host energy.

In eukaryotic cells, LDHB (L-lactate dehydrogenase B chain) converts pyruvate into lactate to generate energy under anaerobic conditions. Lactate metabolism is a common pathway for obtaining energy for bacteria growing under anaerobic conditions [10]. To validate the interaction between DnaK and LDHB, biolayer interferometry (BLI) was used. In the BLI experiments, DnaK was labelled with biotin (21217; Thermo Fisher Scientific) and then immobilized onto Streptavidin (18-5019; Sartorius, Göttingen, Germany) probes. LDHB was diluted at gradient concentrations to interact with the immobilized biotinylated DnaK. ForteBio Octet 96 (ForteBio, Menlo Park, USA) was used to monitor the interaction. As depicted in Figure 2C, the affinity constant between DnaK and LDHB was 21 nM, indicating high-affinity interactions.

To investigate whether DnaK affects the intracellular growth of *Mtb*, macrophages were infected with H37Ra-overexpressing DnaK. First, the ORF of DnaK was cloned and inserted into the pMV261 vector and transfected into H37Ra. The macrophages were prepared by inducing monocyte THP-1 differentiation using PMA. H37Ra with DnaK expression or wild-type H37Ra were cocultured with macrophages at an MOI of 10 for 4 h. The supernatant was discarded to remove the free bacteria. After cultivation for 48 h, the cell lysates were diluted and plated on 7H10 medium. As shown in Figure 2D, DnaK promotes the growth of *Mtb* inside macrophages.

In conclusion, our preliminary findings suggest that DnaK promotes the proliferation of *Mtb* within macrophages by utilizing cellular glycan metabolism. During the latent infection or dormant phase, DnaK may play a role in allowing *Mtb* to adapt to the host environment for long-term survival.

In summary, this study established a PPI network between 21 proteins secreted by *Mtb* and human proteins. Through in-depth analysis of the biological processes associated with these PPIs, we discovered that the *Mtb* protein DnaK significantly upregulates glycan metabolism in human cells. The interaction between DnaK and the host lactate dehydrogenase LDHB was confirmed, and further functional analysis revealed that DnaK enhances *Mtb* survival within macrophages. This study provides potential drug targets for combating *Mtb*.

## Supporting information

24133Table_S2

24133Table_S3

24133Table_S1

24133Table_S4

## Supplementary Data

Supplementary data is available at *Acta Biochimica et Biophysica Sinica* online.
